# Uric Acid Stimulates Fructokinase and Accelerates Fructose Metabolism in the Development of Fatty Liver

**DOI:** 10.1371/journal.pone.0047948

**Published:** 2012-10-24

**Authors:** Miguel A. Lanaspa, Laura G. Sanchez-Lozada, Christina Cicerchi, Nanxing Li, Carlos A. Roncal-Jimenez, Takuji Ishimoto, Myphuong Le, Gabriela E. Garcia, Jeffrey B. Thomas, Christopher J. Rivard, Ana Andres-Hernando, Brandi Hunter, George Schreiner, Bernardo Rodriguez-Iturbe, Yuri Y. Sautin, Richard J. Johnson

**Affiliations:** 1 Division of Renal Diseases and Hypertension, Department of Medicine, University of Colorado Denver, Aurora, Colorado, United States of America; 2 Laboratory of Renal Physiopathology and Nephrology Department, INC Ignacio Chavez, Mexico City, Mexico; 3 Cardero Therapeutics, Incorporated, Menlo Park, California, United States of America; 4 Instituto Venezolano de Investigaciones Científicas-Zulia and Hospital Universitario y Universidad del Zulia, Maracaibo, Venezuela; 5 Division of Nephrology, Hypertension and Transplantation, Department of Medicine, University of Florida, Gainesville, Florida, United States of America; Pennington Biomed Research Center, United States of America

## Abstract

Excessive dietary fructose intake may have an important role in the current epidemics of fatty liver, obesity and diabetes as its intake parallels the development of these syndromes and because it can induce features of metabolic syndrome. The effects of fructose to induce fatty liver, hypertriglyceridemia and insulin resistance, however, vary dramatically among individuals. The first step in fructose metabolism is mediated by fructokinase (KHK), which phosphorylates fructose to fructose-1-phosphate; intracellular uric acid is also generated as a consequence of the transient ATP depletion that occurs during this reaction. Here we show in human hepatocytes that uric acid up-regulates KHK expression thus leading to the amplification of the lipogenic effects of fructose. Inhibition of uric acid production markedly blocked fructose-induced triglyceride accumulation in hepatocytes in vitro and in vivo. The mechanism whereby uric acid stimulates KHK expression involves the activation of the transcription factor ChREBP, which, in turn, results in the transcriptional activation of KHK by binding to a specific sequence within its promoter. Since subjects sensitive to fructose often develop phenotypes associated with hyperuricemia, uric acid may be an underlying factor in sensitizing hepatocytes to fructose metabolism during the development of fatty liver.

## Introduction

Obesity, type 2 diabetes, and non-alcoholic fatty liver disease (NAFLD) are increasing throughout the world [1], [2]. The current epidemics of these conditions have been linked to the increased intake in the last decades of added sugars (primarily in the form of sucrose and high fructose corn syrup, HFCS). A major component of added sugars is fructose, which constitutes 50% of the content of sucrose, and 55% of the most common form of HFCS. Fructose is distinct from glucose in its ability to induce features of metabolic syndrome (insulin resistance, fatty liver, dyslipidemia, and intraabdominal fat accumulation) both in humans [3], [4] and laboratory animals [5]. Of interest, the mechanism whereby fructose induces fatty liver appears to be independent of total energy intake. However, one common finding in clinical studies is that the effect of fructose to induce fatty liver and hypertriglyceridemia varies significantly between humans [6], [7], [8] while the mechanism accounting for these differences in sensitivity to the effects of fructose remains unknown.

One key difference between fructose and glucose is in the initial metabolism. Fructose is metabolized in the liver by fructokinase (ketohexokinase, KHK), which uses ATP to phosphorylate fructose to fructose-1-phosphate. Unlike hexokinases, which phosphorylate glucose and have a negative feedback system to prevent excessive phosphorylation, KHK phosphorylates fructose as rapidly as it can, and this commonly leads to intracellular phosphate depletion. Lower intracellular phosphate levels result in the activation of AMP deaminase, which converts the AMP to IMP, inosine, and eventually uric acid. Uric acid then rises inside the cells and spills out into the circulation [9]. Thus, fructose is distinct from glucose in its ability to cause intracellular phosphate depletion, ATP depletion, and uric acid generation in the liver [10], [11]. Recently our group has shown that intracellular uric acid can induce inflammatory effects and oxidative stress in vascular cells and adipocytes [12], [13], [14].

Exposure to fructose is known to increase KHK expression in hepatocytes of animals [15], [16] and humans [17] thus sensitizing the cells to its metabolic/lipogenic effects. In this manuscript, we studied the mechanisms whereby fructose up-regulates KHK expression in human hepatocytes. Here, we demonstrate that uric acid stimulates the up-regulation of KHK in response to fructose and that blockade of its production by inhibition of xanthine oxidase results in lower KHK levels and amelioration of the lipogenic effects of fructose. Conversely, fructose-induced lipogenesis was significantly increased in hepatocytes pre-exposed to uric acid in a dose-dependent manner. Therefore, the observed differences in responsiveness to fructose in humans could be accounted in part to the role that uric acid plays on the expression of KHK in hepatocytes.

## Materials and Methods

### Methods

See supplemental methods (Methods S1) for more details.

### Ethics Statement

All Animal experiments were performed according to protocols approved by the University of Colorado Animal Care and Use Committee.

### Cell Culture and Silencing

The established human hepatocyte cell line HepG2 was maintained as described elsewhere. Expression of KHK in HepG2 cells was stably silenced. Briefly, lentiviral particles codifying for a silencing sequence were obtained from Open Biosystems (KHK, Hunsville, AL). HepG2 cells previously treated with polybrene (10 µg/ml) were exposed to the lentiviral particles for 24 hours for transduction. After exposure, medium was removed and cells were incubated in normal media in the presence of puromycin (2 µg/ml) to select transducted cells. Clones with greater than 90% silencing as assessed by western blot analysis were selected from colonies growing in plates from a 10-fold dilution series in media prepared with 2 µg/ml puromycin antibiotic. In experiments involving allopurinol, probenecid and C75 treatment, cells were pre-incubated with these compounds for 8 hours prior exposure to fructose or uric acid.

### Rat Experiments

Adult male Sprague-Dawley breeder rats (Charles Rivers, Wilmington, MA) were housed in the animal facility at the University of Colorado. Rats were kept under temperature- and humidity-controlled specific pathogen-free conditions and maintained on a 12 hour light-dark cycle. Animals received normal chow containing 18% protein and 6% fat (3.1 kcal/g of metabolizable energy) (2918, Harlan Laboratories, Madison, WI).The University of Colorado Animal Care and Use Committee approved the experimental protocols. Rats were randomly divided into three groups: Control (n = 6), fructose 10% in drinking water (n = 6) and fructose 10% with allopurinol (30 mg/kg) (n = 6). Rats received fructose for 10 days and water and food consumption was closely monitored. At sacrifice, serum and livers were collected. Liver was immediately processed for oil red O and PAS staining and snap frozen for protein, uric acid and triglyceride determination.

### Immunoprecipitation of Acetylated ChREBP

Levels of acetylated ChREBP in HepG2 cells were determined as described by Bricambert et al [18].

### Chromatin Immunoprecipitation (ChIP) Assay

HepG2 cells were resuspended in PBS to 10^6^ cells/ml. DNA/protein interaction was cross-linked at room temperature for 10 min with paraformaldehyde at a final concentration of 1%. The cross-linking reaction was quenched by addition of glycine, and cells were pelleted and resuspended in lysis buffer containing: 5 mM PIPES, pH 8.0, 85 mM KCl supplemented with 0.5% Nonidet P-40, and mixture protease inhibitor (Roche Applied Science, Indianapolis, IN). The nuclear extract was pelleted by microcentrifugation at 2,000 rpm for 5 min, and genomic DNA was precleared by addition of protein A/G-agarose slurry and further centrifugation. ChrEBP-DNA complexes were pulled down by overnight incubation at 4°C with an anti-ChREBP antibody (Santa Cruz Biotechnology Inc.) and further addition of protein A/G-agarose slurry. Agarose beads were washed twice with PBS supplemented with 1% Nonidet P-40, 0.5% sodium deoxycholate, and 0.1% SDS, followed by four washes in buffer. Agarose beads were collected by centrifugation, and cross-linking was reversed by overnight incubation at 67°C. Relative promoter occupancy was analyzed by real time PCR employing the following primers: Proximal ChoRE forward, 5′TCTGCGTCGACCTGGTCATG-3′ and reverse, 5TTCAGCCGACGTAGACACGT3′, Distal ChoRE forward 5′TGAGGGTGTCTGAACGGT3’ and reverse 5′GAGCTGTTAAGGGCACCCAG3′.

### KHK Luciferase Assays and Site-directed Mutagenesis

The luciferase reporter constructs was engineered from the first 4 kb 5′upstream of the first exon of human KHK by directional cloning employing the following primers: forward 5′CAGGCTAGCTCAATGACTCACTGAATGAG3′ and reverse 5′CAGCTCGAGTCCTCGATCGTTTAGTATGG3′, which was cloned between the *NheI and XhoI* sites (restriction sites in the primers employed are shown underlined above) before the luciferase cassette of the pGL3-Luc vector (Promega, Madison, WI). This construct thus evaluated the importance of the ChoRE sites in the KHK promoter region. Site-directed mutagenesis (Stratagene, La Jolla, CA) was employed following the manufacturer's protocol to modify the ChoRE sites. For the distal site, nucleotides CACGTG were substituted for TTCCCA while for the proximal ChoRE site nucleotides CGCTGT were substituted by TTCTCA. The primers employed were (substitution is shown underlined): Distal ChoRE forward, 5′CTGCGCGAGGGCCTTTCCAGATTCCCAGCTGGGCTCTGGGCT3′ and reverse, 5′AGCCCAGAGCCCAGCTGGGAATCTGGAAAGGCCCTCGCGCAG3′. Proximal ChoRE. Forward 5′TCCTTGTCCCAGGCGTGTTCACAGCGCCACTGGCTGGACGCT3′ and reverse, 5′AGCGTCCAGTGGCGCTGTGAACACGCCTGGGGACAAGGA3′. The luciferase activity was measured in HepG2 cells using the Luciferase Assay System (Promega) according to the manufacturer’s instructions and as described previously [19]. Briefly, HepG2 cells in six-well plates grown to 80% confluence were transiently transfected with each construct and a β-galactosidase reporter (used for normalizing the transfection efficiency). Replicates were incubated at normal conditions or with acute exposure to fructose for 12 h. Cells were then lysed directly in luciferase reporter lysis buffer (Promega). Supernatants from cell lysates were mixed with luciferase substrate and measured immediately with a Luminoskan Ascent DCReady luminometer (Labsystems). All transfection experiments were performed in triplicate. Luciferase activity was normalized to β-galactosidase expression as previously described.

### Statistics and Data Analysis

All data are presented as the mean ± standard error of the mean (SEM). Data graphics and statistical analysis were performed using Instat (version 3.0) and Prism 5 (both Graph Pad Software, San Diego, CA). Data was analyzed for normality tests and using one way ANOVA. Multiple group corrections were performed using the method of Bartlett. In most cases experiments were performed 3 times with independent replicates. Total data points (n) are identified in figure legends. P values <0.05 were recognized as statistically significant.

**Figure 1:**
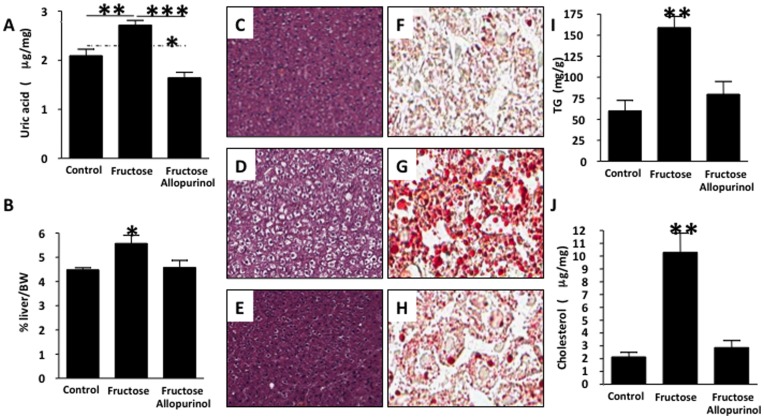
Allopurinol prevents the development of hepatic steatosis in adult male rats drinking fructose. A) Quantity of UA in liver extracts from control, fructose-fed and fructose with allopurinol-fed rats. B) Liver weight normalized to body weight is expressed as percent body weight in control, fructose-fed and fructose with allopurinol-fed rats. Oil red O–stained section of fructose-fed liver. (C–H) Images of rat liver taken using an Aperio Scanscope at × 20 magnification. C–E) H&E-stained section of control (C) fructose (D) and allopurinol fed liver (E). F–H) Oil red O–stained section of control (F), fructose (G) and allopurinol (H) fed liver. I) Quantity of TG in liver extracts from control, fructose-fed and fructose with allopurinol-fed rats. J) Quantity of cholesterol (total and sterol esters) in liver extracts from control, fructose-fed and fructose with allopurinol-fed rats. (n = 5 for each group).

## Results

### Fructose-induced Liver Steatosis is Ameliorated by Lowering Intracellular Uric Acid

Fatty liver in adult male rats was induced with a 15% fructose solution in drinking water for 10 days and compared to a group given fructose plus allopurinol (a xanthine oxidase inhibitor, 30 mg/kg in drinking water) and to control diet alone. Fructose intake was equivalent in rats given fructose and allopurinol and rats given fructose alone (Table S1). Liver uric acid levels were significantly higher in fructose-fed rats compared to control rats with the lowest levels in the allopurinol-treated group (Figure 1A). As a percentage of total body weight, the livers of control, fructose and fructose plus allopurinol rats were 4.5%, 5.6% and 4.5%, respectively (p<0.05; Figure 1B). Fructose-fed rats developed fatty liver, with the hepatocytes diffusely enlarged with clear, sharply bordered cytoplasmic vacuoles (Figure 1D) when compared with control or fructose plus allopurinol treated groups (Figure 1C and 1E). Oil red O staining confirmed minimal lipid content in the control livers (Figure 1F), while fructose-fed livers contained cytoplasmic vacuoles that stained pink with lipids (Figure 1G). Livers from rats receiving allopurinol with fructose showed minimal lipid staining (Figure 1H). To determine which lipid fraction(s) were increased in the fructose-fed livers and therefore blocked with allopurinol, TG and cholesterol content of the liver extracts was measured using a colorimetric assay. TG content was significantly increased in fructose-fed rats versus control and fructose plus allopurinol fed rats (p<0.01, Figure 1I). Cholesterol content was significantly increased in fructose-fed rats and also reduced by allopurinol treatment (p<0.01, Figure 1J). These data show that fructose-induced fatty liver is dependent on xanthine oxidase.

**Figure 2:**
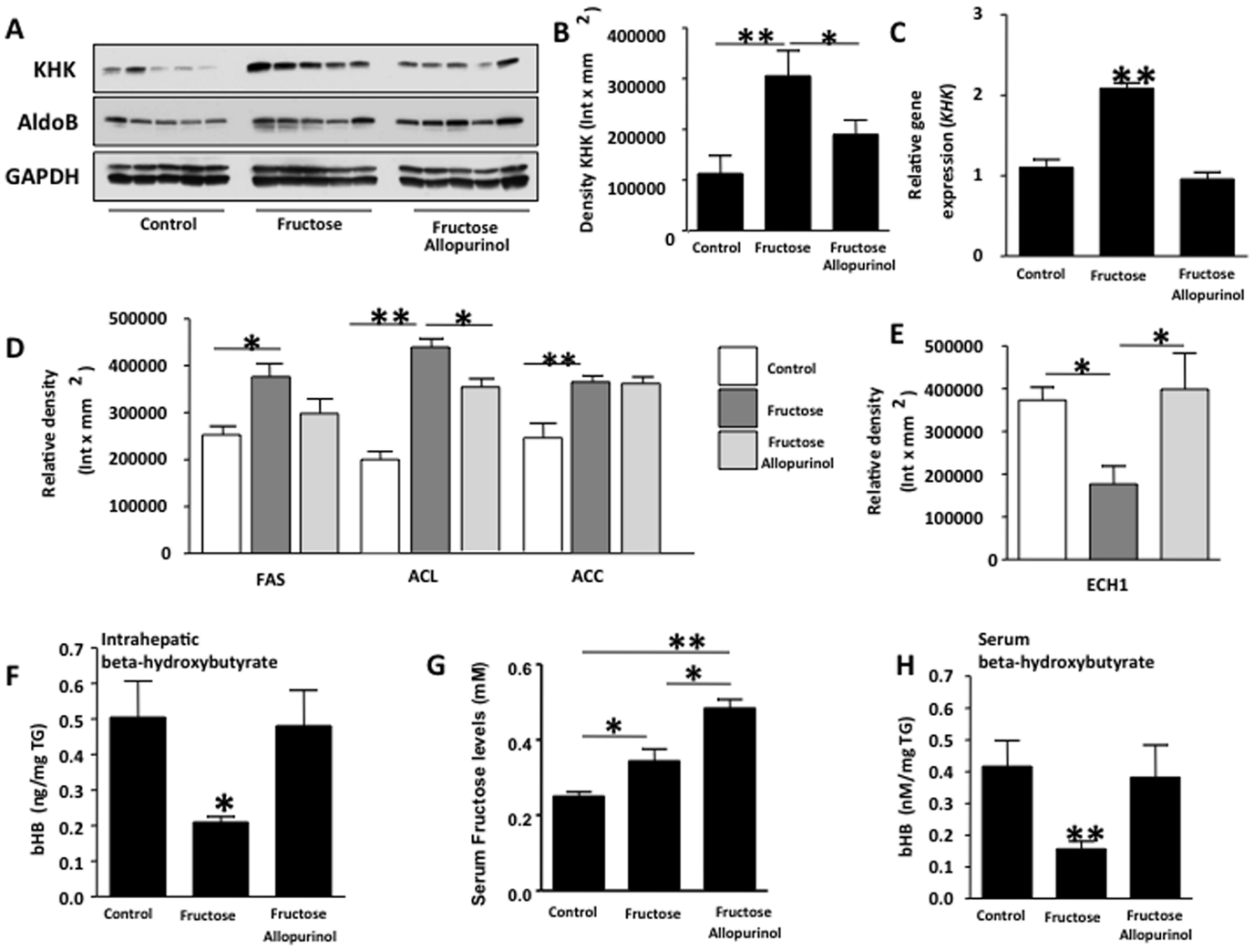
Allopurinol prevents KHK up-regulation in adult male rats drinking fructose (n = 5 for each group). (A–B) KHK and AldoB protein expression in liver extracts from control, fructose-fed and fructose with allopurinol-fed rats. C) mRNA expression of *khk* normalized to *β-actin* levels in liver extracts from control, fructose-fed and fructose with allopurinol-fed rats. D) Expression of lipogenic proteins FAS, ACC and ACL in liver extracts from control, fructose-fed and fructose with allopurinol-fed rats. E) Expression of the fat oxidation-related protein ECH1 in liver extracts from control, fructose-fed and fructose with allopurinol-fed rats. F) Beta-hydroxybutyrate levels in liver and serum normalized to triglyceride levels. G) Serum fructose levels in control, fructose-fed and fructose with allopurinol-fed rats. H) Beta-hydroxybutyrate levels in control, fructose-fed and fructose with allopurinol-fed rats.

### Inhibition of Hepatic Uric Acid Production Prevents Liver KHK Up-regulation in Response to Fructose

To understand the mechanism whereby uric acid production mediates fructose-induced fatty liver, we next examined the expression of KHK and aldolase (AldoB) the first two enzymes involved in fructose metabolism. KHK phosphorylates fructose to fructose-1-phosphate which is further converted to dyhydroxyacetone phosphate and glyceraldehyde by AldoB. Hepatic KHK and AldoB expression were increased 2.7-fold and 1.8-fold, respectively, in fructose-fed rats compared to control rats (Figures 2A–C, p<0.01), consistent with previous studies suggesting that fructose can up-regulate KHK [16]. While the expression of AldoB was not different in fructose plus allopurinol treated rats as compared to fructose-fed rats, fructose-induced up-regulation of KHK was prevented by allopurinol indicating that uric acid may regulate KHK expression in the liver. The changes in KHK protein expression (Figures 2A–B) were also observed at the mRNA level (Figure 2C) suggesting that the lack of KHK up-regulation in fructose/allopurinol fed rats might be controlled at a transcriptional level. These studies show that xanthine oxidase regulates the up-regulation of KHK mRNA and protein in response to fructose.

**Figure 3:**
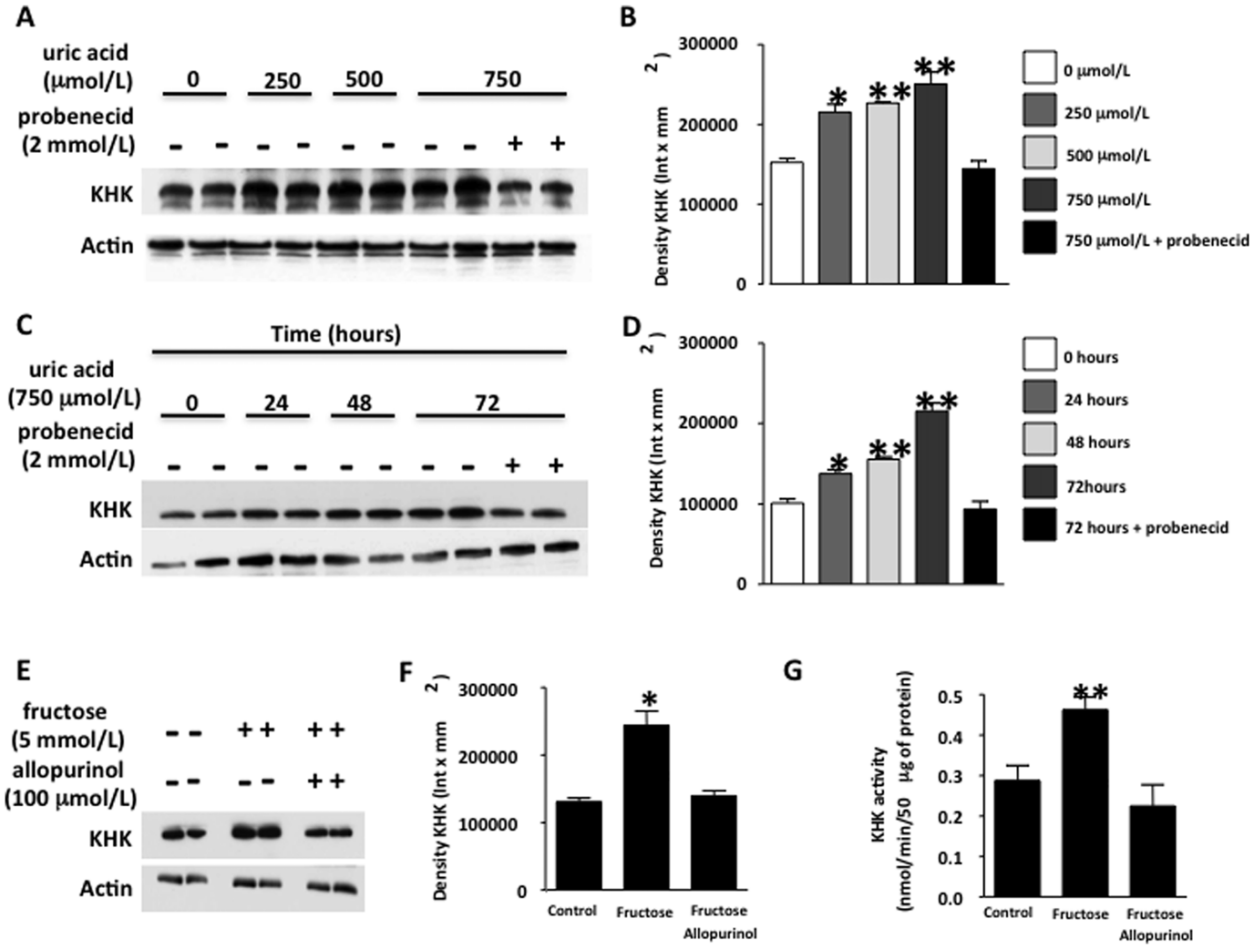
Uric acid up-regulates KHK in human hepatocytes. (A–B) KHK protein expression in cells exposed to increasing levels of uric acid for 72 hours in the presence or absence of the URAT1 inhibitor probenecid (2 mmol/L). (C–D) KHK protein expression in cells exposed to 750 µmol/L uric acid for different time points in the presence or absence of the URAT1 inhibitor probenecid. (E–F) KHK protein expression in cells exposed to 5 mmol/L fructose for 72 hours in the presence or absence of the XO inhibitor allopurinol (100 µmol/L). G) KHK activity in cells exposed to 5 mmol/L fructose for 72 hours in the presence or absence of the XO inhibitor allopurinol (100 µmol/L).

We next asked if lowering intrahepatic uric acid with allopurinol affected the enzymes involved in hepatic lipid accumulation. Since fructose metabolism is associated with increased *de novo* lipogenesis and decreased fat oxidation, we examined the expression of enzymes involved in both fat synthesis (fatty acid synthase (FAS), ATP citrate lyase (ACL) and acetyl-CoA carboxylase (ACC)), as well as fat oxidation (enoyl CoA hydratase-1 (ECH1)). As compared to control rats, fructose slightly but significantly stimulated the hepatic expression of FAS, ACL and ACC (Figure 2D). However, little or no change in expression was observed in fructose/allopurinol treated rats as compared to fructose alone. In contrast, fructose-fed fats had a reduction in hepatic ECH1 expression (53% reduction, p<0.05 versus control) that was significantly prevented by allopurinol indicating that uric acid may participate in the blockade of fat oxidation (Figure 2E). Consistent with the changes in hepatic ECH1 expression, liver and serum β-hydroxybutyrate levels, which are ketoacids generated during fat oxidation, were significantly lower in fructose-fed rats (60.5% and 64.5% reduction, respectively as compared to control) but not in fructose/allopurinol fed rats (Figure 2G and 2H). Consistent with lower hepatic KHK expression in fructose and allopurinol fed rats, we found that serum fructose levels were significantly higher in allopurinol fed rats, perhaps as a consequence of lower fructose metabolism.

**Figure 4:**
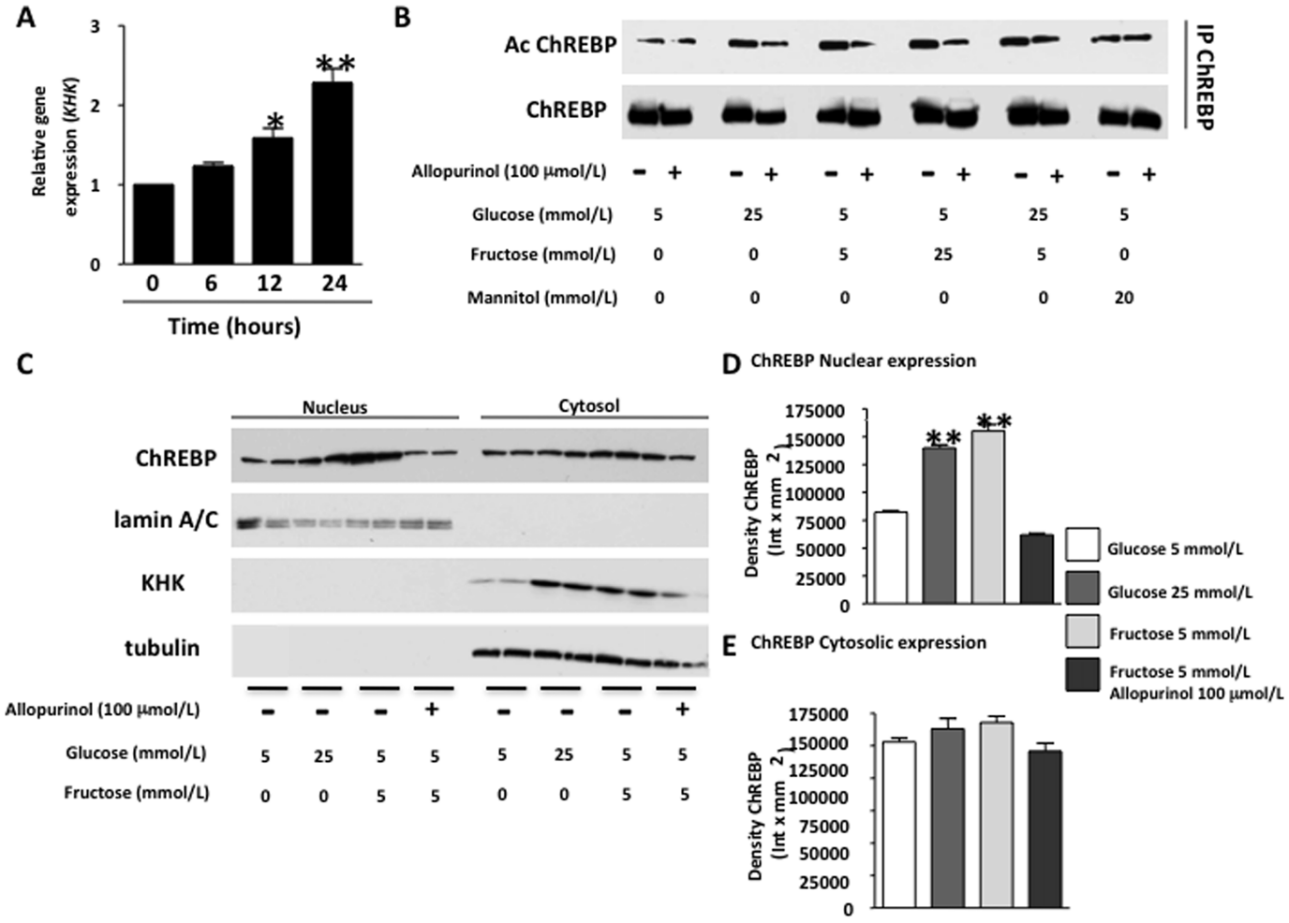
Allopurinol prevents fructose-induced CHREBP acetylation and nuclear translocation in human hepatocytes. A) KHK mRNA expression in cells exposed to fructose (5 mmol/L) for different time points. B) Analysis of acetylation state in immunoprecipitated ChREBP in cells exposed to glucose, fructose or mannitiol in the presence or absence of allopurinol. (C–E) ChREBP and KHK expression in nuclear and cytoplasmic extracts of cells control and incubated with glucose (25 mM) and fructose (5 mM) in the presence or absence of allopurinol.

### Uric Acid Up-regulates KHK Expression in Human Hepatocytes Independently of Fructose

In order to better characterize the role of uric acid in the KHK expression observed in the rats, we used a cell culture system in which uric acid could be added in the absence of fructose. To this end, we employed human hepatocytes (HepG2 cells), which in contrast to rat hepatocytes, lack uricase. We exposed the cells to increasing levels of uric acid, from 0 to 750 µmol/L (4–12 mg/dL) that had previously tested negative for endotoxin and urate crystals (polarized microscopy). As shown in Figure 3A–B, exposure of cells to uric acid for 72 hours resulted in KHK up-regulation in a dose-dependent manner that was significant even at 250 µmol/L (41.2% increase at 250 µmol/L p<0.05 versus control, 48.6% increase at 500 µmol/L p<0.01 versus control and 68.8% increase at 750 µmol/L p<0.01 versus control). KHK up-regulation by uric acid was blocked in the presence of the organic anion transport inhibitor, probenecid (2 mmol/L), which mediates soluble uric acid uptake indicating that uric acid transported inside the cell is responsible for KHK up-regulation. Intracellular uric acid levels are shown in Figure S1. Next, we examined the onset of KHK up-regulation by uric acid (750 µmol/L). KHK protein expression was increased by 35% 24 hours after uric acid exposure (p<0.05) with a more robust increment at 48 and 72 hours (Figure 3C–D). These studies show that uric acid regulates KHK expression in HepG2 cells.

**Figure 5:**
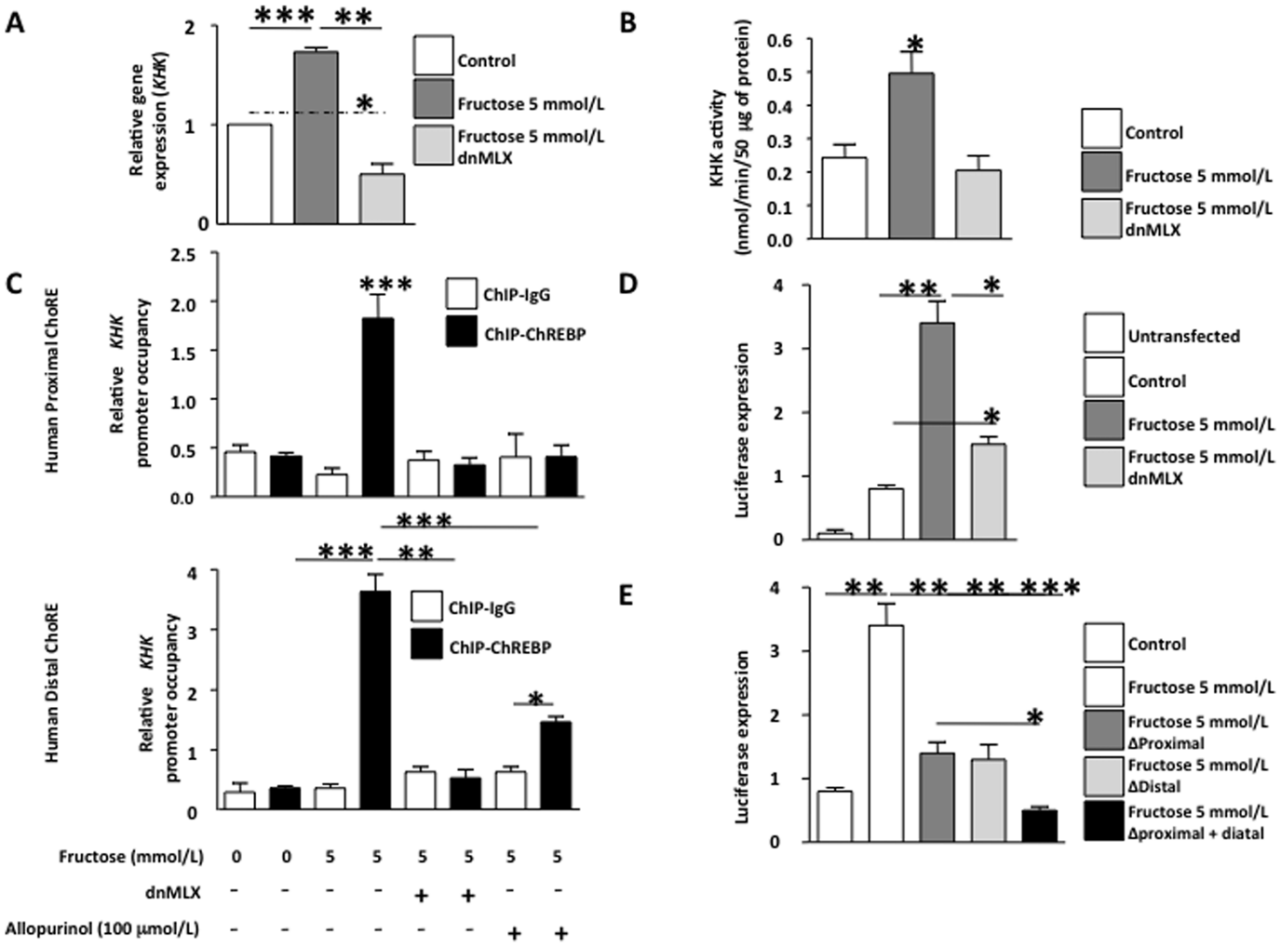
Identification in human hepatocytes of ChoRE sites in *khk* promoter that are activated by fructose and blocked with allopurinol. A) mRNA expression of *khk* in cells exposed to fructose in the presence of ChREBP dominant negative (dnMLX). B) KHK activity in cells exposed to fructose in the presence of ChREBP dominant negative (dnMLX). C) ChIP analysis and *khk* promoter occupancy in distal and proximal ChoRE sites by ChREBP in cells exposed to fructose in the presence of ChREBP dominant negative (dnMLX) or allopurinol. D) Luciferase expression in human hepatocytes transfected with pGL3-*khk*proximal ChoRE and exposed to fructose in the presence of ChREBP dominant negative (dnMLX) E) Luciferase expression in human hepatocytes transfected with pGL3-*khk*distal ChoRE and exposed to fructose in the presence of ChREBP dominant negative (dnMLX) or allopurinol.

We next asked whether fructose-induced up-regulation of KHK was mediated by uric acid. HepG2 cells were exposed to fructose (5 mmol/L) for 72 hours in the presence or absence of allopurinol (100 µmol/L) and KHK expression analyzed by western blot. Intracellular uric acid levels are shown in Figure S1. As shown in Figure 3E–F, KHK expression was significantly up-regulated by fructose alone (p<0.01) but not in the presence of allopurinol indicating that uric acid is responsible for fructose-induced KHK up-regulation. Enzymatic activity of KHK was also lower in cells treated with fructose plus allopurinol compared to fructose alone (p<0.01, Figure 3G).

**Figure 6:**
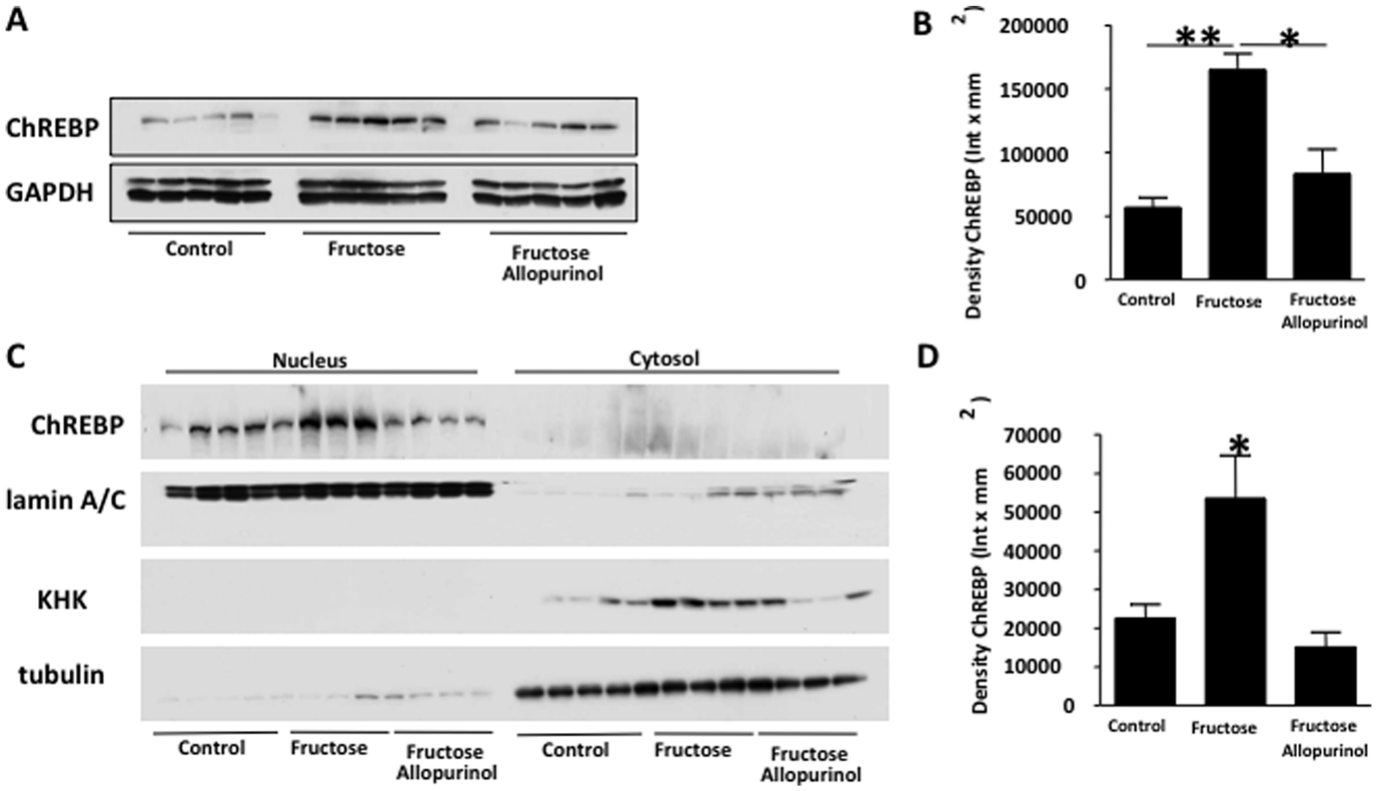
Allopurinol prevents fructose-induced CHREBP nuclear translocation in adult male rats drinking fructose. (A–B) ChREBP protein expression in liver extracts from control, fructose-fed and fructose with allopurinol-fed rats. (C–D) ChREBP and KHK expression in nuclear and cytoplasmic extracts of liver extracts from control, fructose-fed and fructose with allopurinol-fed rats.

### Fructose Stimulates the Transcriptional Activity of ChREBP in the Human KHK Promoter in a Uric Acid-dependent Manner

To determine how uric acid might regulate KHK expression, we examined the transcription factor ChREBP which is known to have KHK as one of its target genes [20]. ChREBP has also been reported to stimulate lipogenesis in response to fructose [21], [22]. Consistent with the data in Figure 2D, we found that KHK mRNA expression was significantly increased by fructose in human hepatocytes (Figure 4A). Next, we examined the role of uric acid in the activation of ChREBP by fructose. Since ChREBP is acetylated in the nucleus in order to be active, we immunoprecipitated ChREBP and determine its acetylation state in control and cells exposed to high glucose (25 mmol/L, positive control) and fructose in the presence or absence of allopurinol (Figure 4B). As shown in the figure, glucose and fructose increased the acetylation state of ChREBP and combination of both sugars did not increase this further. In contrast, mannitol employed at the same molarity as glucose (osmotic control) did not increase ChREBP acetylation. Interestingly, ChREBP acetylation state was significantly reduced not only in fructose- but also in glucose-exposed cells when allopurinol was present indicating that uric acid mediates ChREBP activation. Consistent with increased ChREBP acetylation, both glucose and fructose significantly increased ChREBP nuclear expression (Figure 4C–E). The nuclear localization of ChREBP was reduced by allopurinol suggesting that uric acid acts by stimulating ChREBP nuclear translocation in human hepatocytes rather than activating ChREBP acetylation in the nucleus. Increased nuclear ChREBP expression was paralleled by an up-regulation of KHK in the cytoplasmic fraction. However, we could not observe decreased ChREBP expression in the cytoplasm possibly because ChREBP may be activating its own transcription [20].

**Figure 7:**
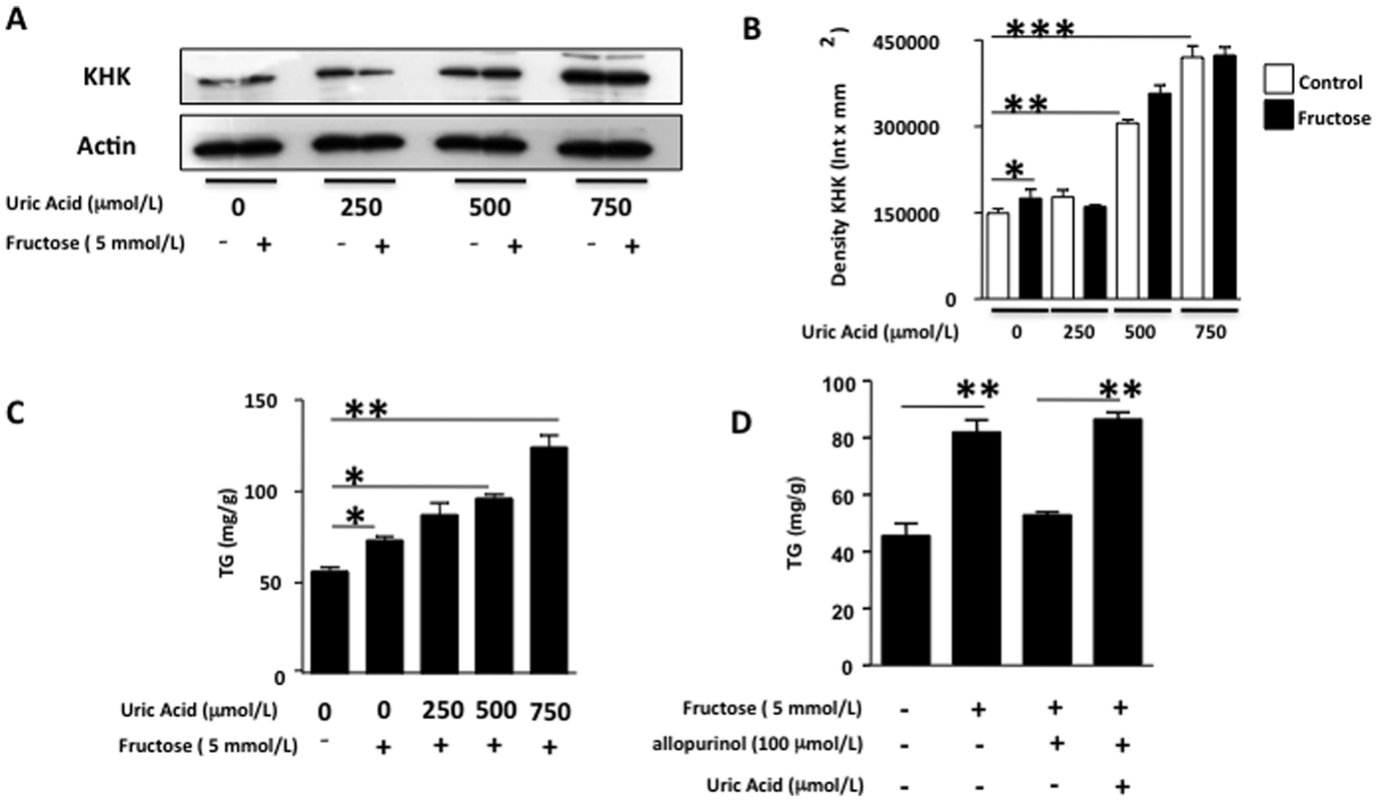
Uric acid sensitizes human hepatocytes to fructose. (A–B) KHK expression in cells pre-exposed to different amounts of uric acid for 72 hours and further incubated with the same amount of fructose for 24 hours. C) Concentration of TG in liver extracts from cells pre-exposed to different amounts of uric acid for 72 hours and further incubated with the same amount of fructose for 24 hours. D) Adding back uric acid reverts the inhibitory effect of allopurinol on TG accumulation in fructose-exposed HepG2 cells.

To determine whether ChREBP activates the transcription of KHK in response to fructose, we transducted hepatocytes with adenoviral particles containing a dominant negative ChREBP (dnMLX). Efficacy of the dominant negative in HepG2 cells was confirmed by exposing the cells to high glucose medium and determining the mRNA expression of well established ChREBP target genes (Figure S2). The up-regulation of KHK mRNA expression in response to fructose was blocked in the presence of dnMLX; indeed, the mRNA levels were significantly lower than cells transducted with control adenoviral particles (53.4% reduction, p<0.05) indicating that ChREBP might control basal expression of KHK (Figure 5A). Consistent with lower KHK expression, KHK activity was also significantly reduced in dnMLX transducted cells (Figure 5B).

Next, we identified two consensus sites within the human KHK promoter –named proximal and distal ChoREs- that resemble the idealistic ChREBP consensus sites consisting of two Ebox sequences separated by five nucleotides (Figure S2B). Human distal ChoRE was found at 2.9 kb upstream of the first exon of human KHK and the sequence is conserved between multiple species. On the other hand, proximal ChoRE, identified at 700 bp upstream of the first exon, was not totally conserved with approximately 30% mismatches between species. In order to determine if ChREBP binds to KHK ChoREs in the presence of fructose, we analyzed ChREBP promoter occupancy using chromatin immunoprecipitation followed by real time PCR employing primers flanking the specific Ebox sites. As shown in Figure 5C, the ChREBP promoter occupancy in the proximal and distal ChoREs was significantly higher in hepatocytes exposed to fructose than in control cells (4.43- and 8.03-fold increase, respectively, p<0.001). Since the over-expression of the dnMLX impairs the DNA binding of ChREBP, we did not observe a significant ChREBP promoter occupancy in the presence of dnMLX in either control (not shown) or fructose-exposed cells. We also did not observe a significant increase in ChREBP promoter occupancy in cells exposed to fructose plus allopurinol, possibly due to reduced ChREBP nuclear expression as previously shown in Figure 4C.

To better characterize the role of KHK ChoREs sites in ChREBP-induced KHK transcriptional activation, we cloned the human KHK promoter upstream a luciferase cassette inserted into the pGL3 vector and both ChoRES were then mutated. Since HepG2 cells are difficult to transfect with nude plasmids, we analyzed transfection efficiency in the cells by co-transfecting a β-galactosidase construct and normalizing luciferase signal to β-galactosidase expression. Luciferase expression in fructose exposed cells was significantly higher than control and dnMLX expressing cells (p<0.05, Figure 5D) indicating that this construct positively reacted to fructose in the medium and that ChREBP plays a key role in KHK transcription. We also found a slight but significant increase in luciferase expression in fructose and dnMLX exposed cells compared to control cells (87% increase, p<0.05) indicating that other transcription factors besides ChREBP may be involved in KHK transcriptional activation by fructose. When the role of each specific ChoREs was analyzed we found that mutation of a single ChoRE was enough to significantly reduce fructose-induced luciferase expression (p<0.01, Figure 5E). Mutation of both sites led to a more prominent reduction (86.8% reduction, p<0.001). These studies document that fructose induced up-regulation of KHK mRNA in HepG2 cells is mediated by uric acid stimulation of ChREBP.

After determining that fructose stimulates ChREBP and that the stimulation could be prevented with allopurinol in human hepatocytes, we characterized ChREBP in the livers of fructose and fructose/allopurinol-fed rats. Expression of total ChREBP (cytosolic and nuclear) was significantly higher in fructose-fed rats compared to control and fructose/allopurinol fed rats (1.9- and 1.1-fold increase, p<0.01 and p<0.05 respectively, Figure 6A–B). As previously determined in HepG2 cells, allopurinol prevented nuclear expression of ChREBP in fructose fed rats with a parallel decrease in cytosolic KHK expression indicating that uric acid up-regulates hepatic KHK *in vivo* possibly by enhancing ChREBP nuclear translocation (Figure 6C–D).

### Uric Acid Sensitizes Human Hepatocytes to Fructose by Up-regulating KHK Expression

To analyze the importance of the relative expression of KHK in fructose-induced lipogenesis, we evaluated the response to fixed amounts of fructose of HepG2 cells control or cells that overexpressed KHK. As shown in Figure S3, overexpression of KHK resulted in significantly increased TG accumulation for the same amount of fructose compared to control cells, indicating that KHK expression determines the overall response of hepatocytes to fructose. After determining the importance of KHK levels in fructose metabolism, we studied if uric acid could potentiate the lipogenic effects of fructose by up-regulating KHK expression. HepG2 cells were preincubated with increasing amounts of uric acid (from 0 to 750 µmol/L) for 72 hours and afterwards exposed to fructose (5 mmol/L) for 24 hours. As previously shown (Figure 3A–B), KHK expression was significantly up-regulated by uric acid in a dose-response manner (Figure 7A–B). Further exposure of fructose for 24 hours resulted in no significant increase in KHK expression as compared to uric acid. While fructose significantly increased TG accumulation in control cells not previously exposed to uric acid (30.3% increase, p<0.05), intracellular TG levels were markedly increased when cells were pre-incubated with uric acid in a dose response manner (55.3% for uric acid 250 µmol/L, p<0.05 versus control and non significant versus fructose alone, 71.4% for uric acid 500 µmol/L, p<0.01 versus control and p<0.05 versus fructose alone, and 122.2% for uric acid 750 µmol/L, p<0.001 versus control and p<0.01 versus fructose alone) indicating that uric acid sensitizes hepatocytes to fructose metabolism (Figure 7C). Furthermore, we reversed allopurinol blockade of fructose-induced TG accumulation by adding back uric acid to the cells indicating that the mechanism whereby allopurinol blocks lipogenesis is by reducing intracellular uric acid levels (Figure 7D).

The mechanism whereby fructose induces fat accumulation is thought to be induced by activating de novo lipogenesis. This activation is mediated by the ability of fructose to be metabolized to Acetyl-CoA and glyceraldehyde, the substrates for fatty acid synthase (FAS). In this regard, we have confirmed that blocking FAS activity with the FAS inhibitor, C75 (10 µmol/L), blocks fructose-induced TG accumulation thus validating the lipogenic effects of fructose (Figure S4A). Consistent with a blockade of FAS activity, intracellular acetyl-CoA levels were increased in C75 and fructose-exposed cells (Figure S4B). To determine if the mechanism whereby allopurinol blocks fructose-induced TG accumulation was associated with decreased de novo lipogenesis, we analyzed intracellular levels of acetyl-CoA in C75 treated cells (to prevent Acetyl-CoA metabolism) exposed to fructose alone or in the presence of allopurinol. As shown in Figure S4C, allopurinol significantly reduced acetyl-CoA accumulation in fructose-exposed cells thus confirming that the major mechanism whereby uric acid potentiates fructose response is by increasing acetyl-CoA levels and de novo lipogenesis rates.

## Discussion

The novel finding in this study relate to the discovery for a causal role for uric acid in fructose-induced fat accumulation in the liver. It is known that animals fed fructose show an increased expression of both the fructose transporter, Glut 5, and of fructokinase (KHK) [16]. Here we show that KHK up-regulation by fructose is mediated by the intracellular production of uric acid. Incubation of human hepatocytes with uric acid resulted in KHK protein over-expression in a dose dependent manner which was prevented by addition of the organic anion transport inhibitor, probenecid. Furthermore, we found that KHK is a target gene of the transcription factor associated with carbohydrate metabolism, ChREBP, and that allopurinol could block ChREBP nuclear translocation and activation.

There is increasing evidence that uric acid has a role in hepatic steatosis. First, an elevated serum uric acid is commonly elevated in subjects with nonalcoholic fatty liver disease both in cross-sectional and longitudinal studies [14], [23]–[26]. An elevated uric acid also correlated with histological liver damage in subjects with non-alcoholic fatty liver disease [27]. Studies by Ouyang et al have also linked the hyperuricemia and hepatic steatosis with increased fructose consumption from soft drinks in association with increased fructokinase expression in the liver [17]. Furthermore, subjects with non-alcoholic fatty liver disease who have a history of greater fructose exposure, or who have higher elevated serum uric acid, show a greater hepatic ATP depletion in response to fructose than those with lower baseline uric acid levels [28]. Finally, a single report by Xu et al found that lowering uric acid with allopurinol could reduce hepatic steatosis in the desert sand rat [29]. These studies are consistent with uric acid having a contributory role in hepatic steatosis by up-regulating fructokinase expression and fructose metabolism.

These studies could potentially explain why subjects with hyperinsulinemia show an enhanced metabolic response to fructose [30]–[33]. Insulin resistance is strongly associated with hyperuricemia [34]. It also may explain why male subjects are typically more sensitive to the effects of fructose than female subjects [35], as males have higher uric acid levels. It also provides a mechanism for why lowering uric acid has been found to block fructose-induced metabolic syndrome in rats [5].

One interesting finding was that allopurinol was able to block both fructose and glucose stimulation of ChREBP. Recently we have found that glucose can be converted to fructose inside cells via the polyol pathway, an accessory route consisting of two enzymes, aldose reductase which converts glucose to sorbitol and sorbitol dehydrogenase that metabolizes it to fructose [36]. It is possible that the mechanism by which allopurinol protected against glucose-induced ChREBP activation could involve the intracellular generation of fructose with its subsequent metabolism and generation of uric acid as a byproduct. The mechanism whereby allopurinol blocks ChREBP nuclear translocation remains unknown. We speculate this could be mediated by the inactivation of AMP kinase, a known inhibitor of ChREBP activity [37], [38]. In this regard, we have recently shown that uric acid down-regulates AMPK activation, as determined by its phosphorylation at threonine 172, in fructose-exposed human hepatocytes (M Lanaspa, data unpublished).

It is recognized that serum uric acid can be modulated by a variety of means, including diet, renal function, and even insulin resistance itself [39], [40]. The commonly observed elevations of uric acid in a wide variety of conditions, such as obesity, metabolic syndrome, hypertension and kidney disease has made it difficult epidemiologically to understand its relative importance. More recently, however, it has become evident that soluble uric acid is biologically active and can stimulate the production of inflammatory and vasoactive mediators [41], [42], [43]. This study provides the first evidence that uric acid can directly regulate fructose metabolism.

The observation that uric acid can amplify the lipogenic effects of fructose may also be of relevance to primate evolution. Indeed, it is known that the mutation of uricase, an enzyme that converts uric acid to allantoin, occurred during the mid Miocene at a time when the hominoids in Europe were faced with food shortage due to reduced availability of fruit during the cooler seasonal periods [44]. We have postulated that this mutation may have provided a natural selection advantage by amplifying the ability of fructose to increase fat stores [44]. Since most mammals posses an active uricase enzyme, it could be argued from the results derived of this manuscript that these animals may be protected from fructose effects. However, we found that fructose significantly reduced uricase activity in the liver (Figure S5). We do not know how uricase activity is down-regulated by fructose but since allopurinol-treated rats have normal uricase activity we believe that uric acid itself or rather a metabolite of uric acid degradation by uricase (i.e.: allantoin) may have a negative effect on uricase activity in mammals.

In conclusion, the present study shows that the metabolism of fructose by fructokinase results in the intracellular production of uric acid that feeds back to up-regulate fructokinase and increase the sensitivity of the cell to the triglyceride-raising effects of fructose. Of interest, the blockade of intracellular uric acid with allopurinol could inhibit the effect of fructose to increase fat accumulation. The significant reduction in fat accumulation raises the possibility that uric acid may have additional mechanisms whereby it may control fat accumulation. Studies are ongoing in our laboratory to identify potential mechanisms.

## Supporting Information

Figure S1 **Exposure of human hepatocytes to uric acid results in higher intracellular uric acid levels.** A) Concentration of UA in cell extracts from control and to increasing levels of uric acid exposed cells (from 250 to 750 µmol/L). B) Concentration of UA in cell extracts from control and uric acid exposed cells (750 µmol/L) for 24, 48 and 72 hours. C) Concentration of UA in cell extracts from control, fructose (5 mmol/L) and fructose and allopurinol (100 µmol/L) exposed cells.
(TIF)

Figure S2 **Identification of potential ChoREs sites within the human KHK promoter.** A) Effects of dnMLX in the mRNA response of PKL (Pyruvate kinase-liver specific) to high glucose levels. B) Sequencing of both putative proximal and distal ChoRES identified in the KHK promoter in multiple species incuding humans and primates.
(TIF)

Figure S3 **KHK expression modulates the metabolic response of HepG2 cells to fructose.** A) Representative western blot of a HepG2 cell line overexpressing KHK. Lane 1: mouse liver control, lane 2: mouse KHK knockout control, lane 3 HepG2 control, 4 HepG2 overexpressing KHK (oxKHK). B) TG accumulation of control and oxKHK cells in response to fixed amounts of fructose for 72 hours. *p<0.05, **p<0.01.
(TIFF)

Figure S4 **Allopurinol reduces fructose-induced TG accumulation by decreasing lipogenesis.** A) Inhibition of fatty acid synthase (FAS) activity with C75 (10 µmol/L) blocks fructose-induced TG accumulation in HepG2 cels. B) Inhibition of fatty acid synthase (FAS) activity with C75 further increases intracellular levels of Acetyl-CoA. C) Allopurinol (100 µmol/L) significantly decreases fructose-induced increased levels of intracellular Acetyl-CoA. *p<0.05, **p<0.01.
(TIFF)

Figure S5 **Fructose inhibits uricase activity in rats.** A) Uricase activity assay demonstrating lower activity in fructose-fed rats (F) as compared to control (C, tap water) or fructose and allopurinol (F+AP) drinking rats. *p<0.05 versus rest of the groups.
(TIFF)

Table S1 **Overall and serum parameters of adult male rats drinking fructose with or without allopurinol.**
(DOC)

Methods S1 **Methodology included as supplemental data.**
(DOC)
