# Supplementary figures and images for: Uric Acid Stimulates Fructokinase and Accelerates Fructose Metabolism in the Development of Fatty Liver
Source: PLoS One. 2012 Oct 24;7(10):e47948. doi: 10.1371/journal.pone.0047948 (PMC3480441; doi:10.1371/journal.pone.0047948)

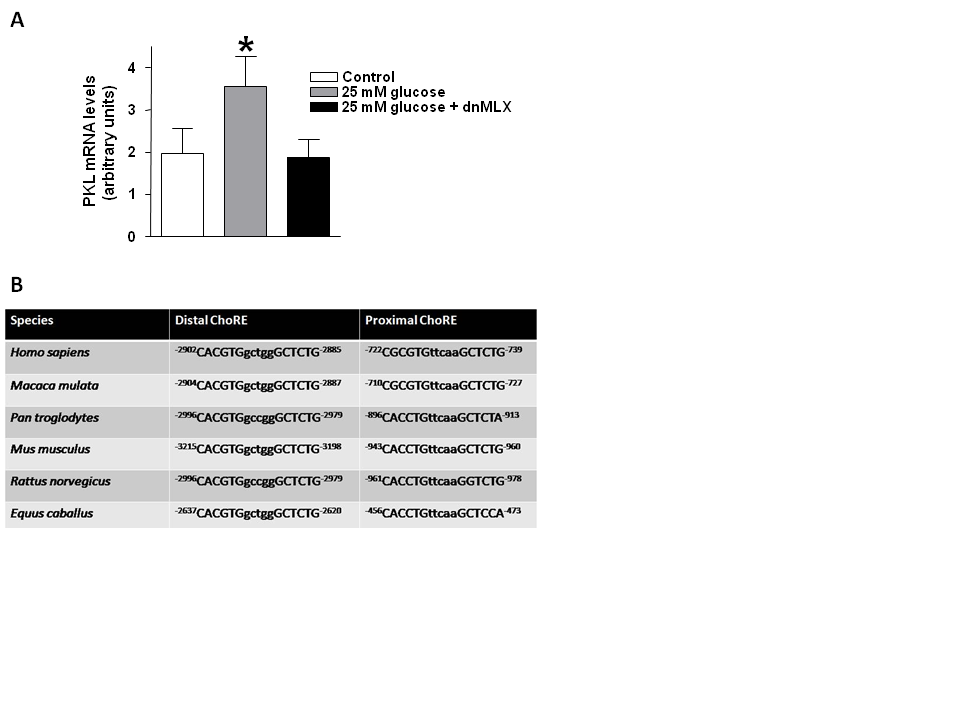

Supplement: Figure S2 — Identification of potential ChoREs sites within the human KHK promoter. A) Effects of dnMLX in the mRNA response of PKL (Pyruvate kinase-liver specific) to high glucose levels. B) Sequencing of both putative proximal and distal ChoRES identified in the KHK promoter in multiple species incuding humans and primates. (TIF) [file pone.0047948.s002.tif]

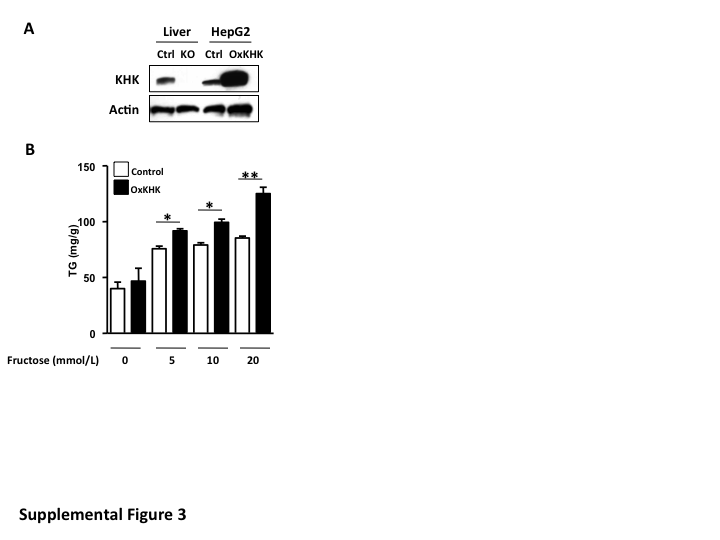

Supplement: Figure S3 — KHK expression modulates the metabolic response of HepG2 cells to fructose. A) Representative western blot of a HepG2 cell line overexpressing KHK. Lane 1: mouse liver control, lane 2: mouse KHK knockout control, lane 3 HepG2 control, 4 HepG2 overexpressing KHK (oxKHK). B) TG accumulation of control and oxKHK cells in response to fixed amounts of fructose for 72 hours. *p<0.05, **p<0.01. (TIFF) [file pone.0047948.s003.tif]

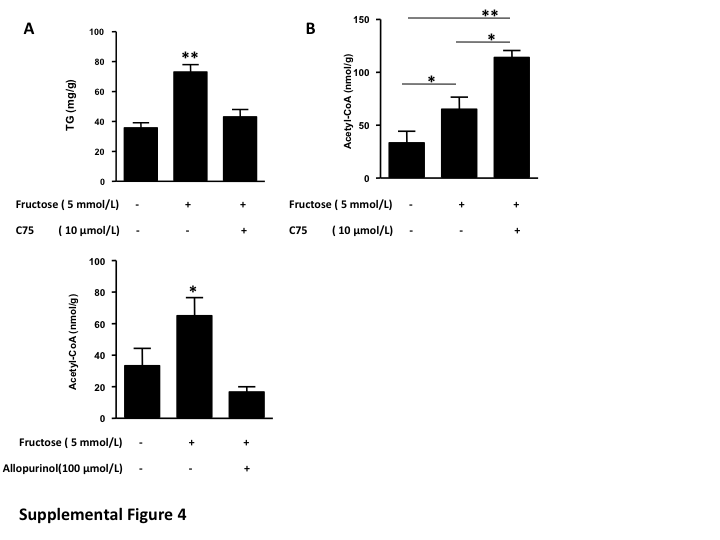

Supplement: Figure S4 — Allopurinol reduces fructose-induced TG accumulation by decreasing lipogenesis. A) Inhibition of fatty acid synthase (FAS) activity with C75 (10 µmol/L) blocks fructose-induced TG accumulation in HepG2 cels. B) Inhibition of fatty acid synthase (FAS) activity with C75 further increases intracellular levels of Acetyl-CoA. C) Allopurinol (100 µmol/L) significantly decreases fructose-induced increased levels of intracellular Acetyl-CoA. *p<0.05, **p<0.01. (TIFF) [file pone.0047948.s004.tif]

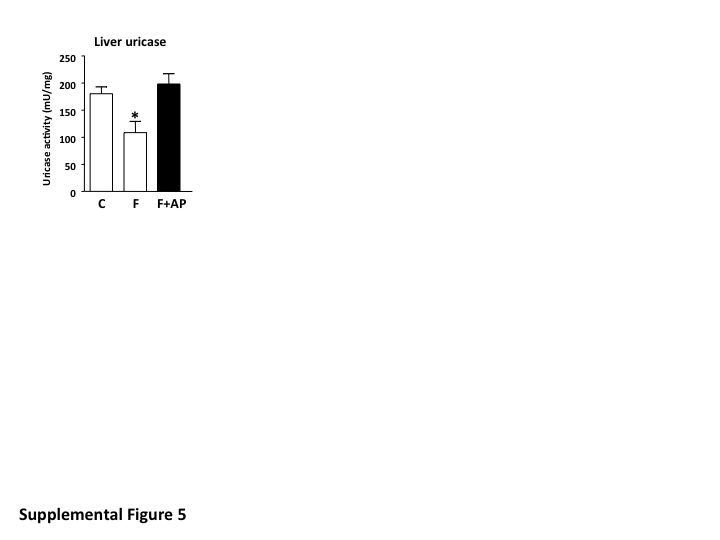

Supplement: Figure S5 — Fructose inhibits uricase activity in rats. A) Uricase activity assay demonstrating lower activity in fructose-fed rats (F) as compared to control (C, tap water) or fructose and allopurinol (F+AP) drinking rats. *p<0.05 versus rest of the groups. (TIFF) [file pone.0047948.s005.tif]
