# Supplementary material for: Uric Acid Stimulates Fructokinase and Accelerates Fructose Metabolism in the Development of Fatty Liver
Source: PLoS One. 2012 Oct 24;7(10):e47948. doi: 10.1371/journal.pone.0047948 (PMC3480441; doi:10.1371/journal.pone.0047948)
Supplement: Table S1 — Overall and serum parameters of adult male rats drinking fructose with or without allopurinol. (DOC) [file pone.0047948.s006.doc]

**Control fructose Fructose + allopurinol ANOVA**

**(n = 6) (n = 6) (n = 6)**

**Body weight (BW) (g) 333.7± 11.63 339.8 ±11.74 329.1 ± 8.91 N.S.**

**Water consumption (ml/day) 32.5± 4.62 72.0 ±14.49^*^ 77.6± 6.80^*^ ^*^p<0.01 vs control**

**Food consumption (g/day) 35.4± 2.07 23.3 ±3.32^*^ 23.8±4.86^*^ ^*^p<0.01 vs control**

**Calorie intake (kcal/day) 116.8±4.27 120.1 ±7.94 125.1±8.56 N.S.**

**BUN (mg/dl) 16.7 ± 1.5 11.0 ± 2.9^*^ 10.1 ± 2.7^*^ ^*^p<0.01 vs control**

**Creatinine (mg/dl) 0.42 ± 0.05 0.44 ± 0.05 0.46 ± 0.05 N.S.**

**Liver/ BW ratio (%) 4.48 ± 0.18 5.57 ± 0.67^*^ 4.47 ± 0.58 ^*^p<0.05 vs rest of columns**

**AST (IU/l) 36.0 ± 1.2 32.4 ± 10.2 41.1 ± 5.5 N.S.**

**ALT (IU/l) 22.6 ± 1.6 21.9 ± 0.6 23.6 ± 1.8 N.S.**

**Serum Uric acid (mg/dl) 1.85 ± 0.1 2.12 ± 0.1^*^ 1.13 ± 0.05^$&^ ^*&^p<0.01and p<0.001 vs control**

**^$^p<0.001 vs fructose**

**Serum Glucose (mg/dl) 151.3 ± 17.1 172.8 ± 47.7 155.4 ± 20.11 N.S.**

**Serum Triglyceride (mg/dl) 163.0 ± 15.5 280.2 ± 74.7^*^ 189.0 ± 24.78 ^*^p<0.05 vs rest of columns**

**Serum Total cholesterol (mg/dl) 91.3 ± 14.2 98.0 ± 26.3 87.8 ± 10.5 N.S.**

**ALT, alanine aminotransferase; AST, aspartate aminotransferase; BUN, blood urea nitrogen. N.S., not significant.**
