# Supplementary material for: Uric Acid Stimulates Fructokinase and Accelerates Fructose Metabolism in the Development of Fatty Liver
Source: PLoS One. 2012 Oct 24;7(10):e47948. doi: 10.1371/journal.pone.0047948 (PMC3480441; doi:10.1371/journal.pone.0047948)
Supplement: Methods S1 — Methodology included as supplemental data. (DOC) [file pone.0047948.s007.doc]

**Supplemental methods:**

***Materials.*** Cell culture medium (RPMI-1640), fetal calf serum, and antibiotics were from Gibco (Rockville, MD). Rabbit polyclonal antibody to KHK (HPA00740) was purchased from Sigma (St Louis, MO), antibodies to NOX4 (SC-30141) and Lamin A (SC-20681) were from Santa Cruz Biotechnology Inc. (Santa Cruz, CA), antibodies to β-actin (3700) and FAS (3180) were from Cell Signaling (Danvers, MA) while antibodies to aldolase B (H00000229-B01) was from Novus Biologicals (Littleton, CO). Secondary antibodies conjugated with horseradish peroxidase were from Cell Signaling and those conjugated with Alexa dyes were purchased from Molecular Probes (Eugene, Orgegon). Fructose, glucose, mannitol, uric acid, and allopurinol were purchased from Sigma while probenecid was obtained from Molecular Probes.

***Determination of intracellular, intrahepatic and serum uric acid and triglycerides*** Cell lysates obtained with MAP kinase lysis buffer as well as serum collected from 8‐hour fasting rats were analyzed using a VetAce autoanalyzer (Alfa Wassermann , West Caldwell, NJ) as previously described (*3*). For triglyceride determination in liver and HepG2 cells, fat was solubilized by homogenization in 1 ml solution containing 5% nonidet P40 (NP‐40) in water, slowly samples were exposed to 80‐100°C in a water bath for 2‐5 minutes until theNP‐40 became cloudy, then cooled down to room temperature. Samples were then centrifuged for 2 minutes to remove any insoluble material. Triglyceride determination with the VetAce autoanalyzer consisted in their initial breakdown into fatty acids and glycerol. Glycerol is then oxidized to generate a product which reacts with the probe to generate color at 570 nm. Similarly, uric acid determination is based in the conversion of uric acid to allantoin, hydrogen peroxide (H2O2) and carbon dioxide by uricase. The H2O2 then, is determined by its reaction with the probe to generate color at approximately 571 nm. Values obtained were normalized per mg of soluble protein in the lysates. Since rat livers contained endogenous uricase, intrahepatic uric acid in liver samples were analyzed employing the quantichrom uric acid determination kit (bioassay systems, Hayward, CA) which is a non‐uricase based system.

***Determination of intracellular acetyl-CoA levels.*** Acetyl-CoA was measured from cell lysates prepared as described previously and Acetyl-CoA determination was evaluated with a commercial kit as per manufacturer’s protocol (K317 Biovision,Milpitas, CA).

***Liver oil red o staining*** Liver tissue collected under isoflurane anesthesia was embedded in Optimal Cutting Temperature gel (OCT; Sakura Finetek, Torrance, CA) and frozen in liquid nitrogen. Air‐dried cryostat tissue sections (8 *μ*m) were dipped in formalin, washed with running tap water, rinsed with 60% isopropanol, and stained with oil red O counterstained with hematoxylin. Macrovesicular fat deposition was defined as the presence of lipid vacuoles that are larger than the nucleus and usually displaces it to the periphery of the cell.

***Protein extraction and Western blotting.*** Protein lysates were prepared from confluent cell cultures and rat tissue employing MAP Kinase lysis buffer as previously described (10). Sample protein content was determined by the BCA protein assay (Pierce). 50 μg of total protein was loaded per lane for SDS-PAGE (10% w/v) analysis and then transferred to PVDF membranes. Membranes were incubated with primary antibodies and visualized using a horseradish peroxidase secondary antibody and the HRP Immunstar® detection kit (Bio-Rad, Hercules, CA). Chemiluminescence was recorded with an Image Station 440CF and results analyzed with the 1D Image Software (Kodak Digital Science, Rochester, NY).

***RNA extraction, analysis and message quantification.*** Cytosolic RNA was isolated from confluent HepG2 cultures using the RNeasy kit (Qiagen, Valencia, CA). RNA was converted to cDNA using the iScript Reverse Transcriptase kit (Bio-Rad, Hercules, CA) as described by the manufacturer. Quantitative PCR primers specific to KHK, PKL and β-actin were designed using Beacon Designer 5.0 software (Premier Biosoft International, Palo Alto, CA). Quantitative PCR for KHK was performed using 5’ACGAGCCTGCCAGATGTG3’ forward primer, and 5’GGTGTTGTGTGCGTCTATCC3’ reverse primer (70 nM each) using a SYBR green master mix (JumpStart® Taq Readymix®, Sigma) on a BioRad I-Cycler. QPCR runs were analyzed by agarose gel electrophoresis and melt curve to verify the correct amplicon was produced. β-actin RNA was used as internal control and the amount of RNA was calculated by the comparative CT method as recommended by the manufacturer.

***Nuclear isolation from HepG2 cells*** Nuclei were purified with the NE-PER kit from Pierce following manufacturer’s protocol. Quantization of nuclear protein was performed by the BCA assay. Lamin A/C expression was employed for equally nuclear loading control by western blot analysis.

***Determination of KHK activity in human hepatocytes.*** KHK activity was determined by the method developed by Hers et al. Briefly, lysates (50 μg) were dissolved in buffer containing 50mM imidazole, 1.85 mM fructose, 4mM NaATP; 4mM MgCL2; 1M KAc, 20mM N-acetylglucosamine, to inhibit the phosphorylation of fructose by hexokinase and 40 mM NaF. The mixture is incubated for 16 minutes at 37°C and stopped by addition of 0.15M ZnSO_4_ and 0.1M Ba(OH)_2_.  Samples were then centrifuged and the residual fructose in the supernatant is measured by addition of 0.1 % alcoholic resorcinol in 30 % HCl and absorbance is read at 515 nm with a multimode plate reader. To calculate absolute values, a standard curve of fructose –from 1μM to 2 mM- is run and analyzed in parallel.

***Overexpression of KHK in HepG2 cells.*** To overexpress KHK in HepG2 cells, the coding sequence was obtained from HepG2 cDNA and inserted into the pLVXIRES puro vector by direct cloning between the EcoRI and NotI sites of the multicloning site. Correct cloning was confirmed by sequencing analysis. Seven micrograms of vector at 1 μg/μl was combined with the Lenti-X HTX packaging system (clontech) and HEK-293T cells maintained in tetracycline free medium were transfected with the components. Medium was collected at 48 hours and efficient production of lentiviral particles was assessed with the Lenti-X Gostix (clontech). HepG2 cells were exposed to the virus and stable clones generated with puromycin. Controls refer to puromycin selected clones transducted with lentiviral particles containing empty pLVXIRES Puro vector.
